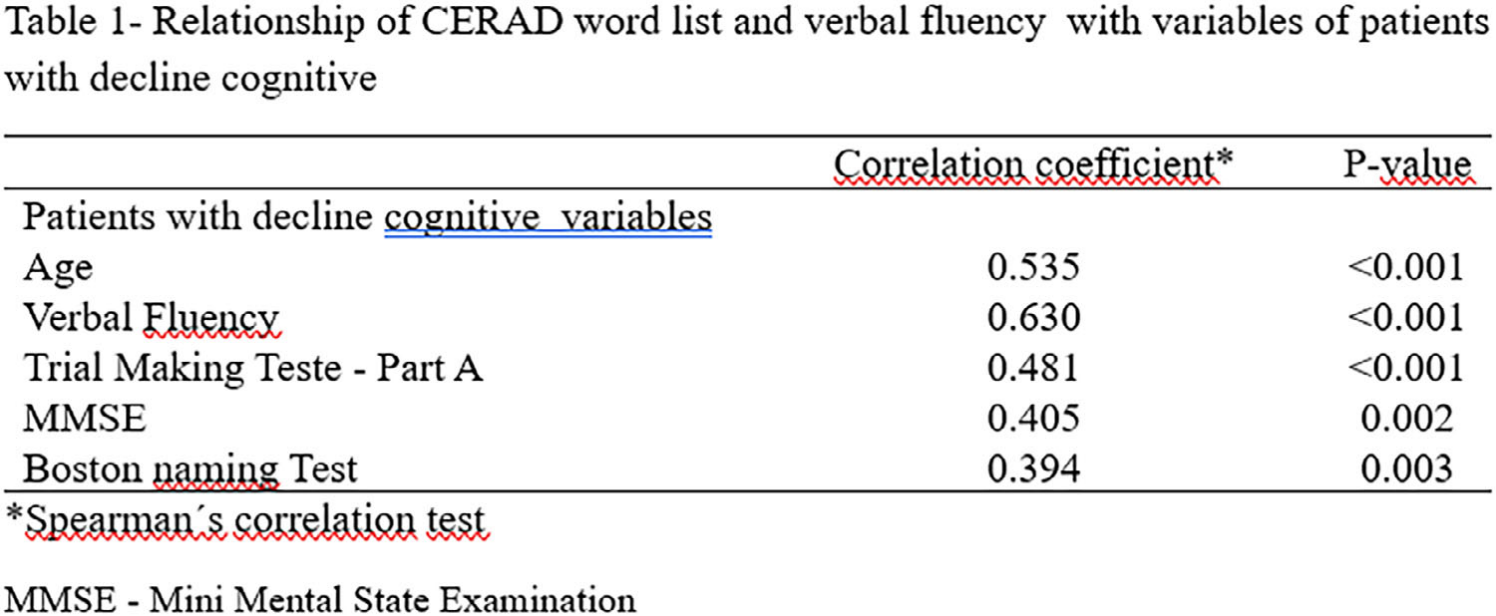# Post‐COVID‐19 cognitive decline: Preliminary findings from a Brazilian cohort study

**DOI:** 10.1002/alz70857_107347

**Published:** 2025-12-24

**Authors:** Glenda Dias dos Santos, Orestes Vicente Forlenza, HCFMUSP COVID‐19 Study Group

**Affiliations:** ^1^ Institute of Psychiatry, Faculty of Medicine, University of Sao Paulo, Sao Paulo, Brazil; ^2^ Centro de Neurociências Translacionais (CNT), Faculdade de Medicina da Universidade de São Paulo, São Paulo, São Paulo, Brazil; ^3^ Laboratory of Neurosciences (LIM27), Departamento e Instituto de Psiquiatria, Hospital das Clínicas, Faculdade de Medicina da Universidade de São Paulo, São Paulo, São Paulo, Brazil

## Abstract

**Background:**

Many people who have been affected by COVID‐19 report impaired cognitive abilities resulting in functional impairments and worsening quality of life. The present study aims to investigate the occurrence of cognitive impairments in a cohort of survivors of moderate or severe forms of COVID‐19.

**Method:**

361 adults were evaluated 24 months after hospital discharge with a cognitive battery involving the tests: Mini Mental State Examination, CERAD battery, Trail Making Test part A and B, and Digit Span.

**Result:**

Of the total sample, 54 adults (15%) showed cognitive changes in the tests, presenting an average of 21 points in the Mini Mental State Examination (SD 2.4). In the CERAD battery, 48.1% (*n* = 26) showed low performance in the verbal fluency test – animal category remembering less than 13 words; 74% (*n* =  40) got less than 13 pictures correct in the Boston naming test; and 83.3% (*n* =  45) recalled less than 6 words in the word list. In the Trail Making Test, part A the average was 115 seconds (SD 61.6) in part B the average was 204.11 seconds (SD 104.5), with 66.7% (*n* = 36) unable to do it the test. In the digit span test, the greatest difficulty was in the indirect order in which 53.7% (*n* = 29) scored 2 points, with an average of 2.39 (SD 1.2). Between the tests, there was a correlation between the CERAD word list and verbal fluency (*r* = 0.630, *p* <0.001), trails A (*r* =  ‐0.481, *p* <0.001), age (*r* =  ‐0.535, *p* <0.001), MMSE (*r* =  0.405, *p* = 0.002) and Boston naming test (*r* = 0.394, *p* = 0.003).

**Conclusion:**

The cognitive battery used was able to identify cognitive deficits related to executive function, memory, attention and language. Identifying patterns of cognitive deficits associated with COVID‐19 is necessary to distinguish cognitive impairments associated with COVID‐19 from other dementias.